# High‐Performance Polymer‐derived Ceramics in LCD 3D Printing

**DOI:** 10.1002/advs.202416176

**Published:** 2025-03-17

**Authors:** H. Yazdani Sarvestani, V. Karamzadeh, A. Kulkarni, A. Sohrabi‐Kashani, T. Lacelle, M.B. Jakubinek, B. Ashrafi

**Affiliations:** ^1^ Aerospace Manufacturing Technology Centre National Research Council Canada Montreal QC H3T 2B2 Canada; ^2^ Division of Emerging Technologies National Research Council Canada Ottawa ON K1A 0R6 Canada

**Keywords:** high‐temperature pyrolysis, LCD 3D printing, mechanical properties, microstructural characterization, polymer‐derived ceramics

## Abstract

This study demonstrates the fabrication of high‐strength, lightweight polymer‐derived ceramics (PDCs) using silicon oxycarbide (SiOC)‐precursor formulations with liquid crystal display (LCD) vat photopolymerization (VPP) technology. Complex geometries, such as gyroids and stochastic lattices, are successfully 3D‐printed and evaluated under varying feature thicknesses and pyrolysis temperatures (800 °C and 1200 °C). Photorheology and thermogravimetric analysis (TGA) validated the efficient curing and pyrolysis characteristics of a printable precursor formulation based on vinyl methoxysiloxane homopolymer (VMM‐010), which demonstrated rapid curing, low viscosity, and compatibility with LCD 3D printing, ensuring precise layering and efficient resin removal. Micro‐CT scans confirmed its structural integrity and absence of voids, even in relatively thick components (≈3 mm). The VMM‐based PDC lattices achieved specific compressive strengths up to 9.4 MPa g⁻¹ cm^3^, a 50‐fold improvement over comparable lattices produced with a high‐porosity SiOC PDC, and exceptional high‐temperature stability, maintaining structural integrity after 2 h at 1500 °C. Compositional analysis revealed lower free carbon content and improved ceramic phase formation, driving the enhanced mechanical and thermal performance of the VMM‐based ceramic. These findings underscore the scalability, reliability, and superior performance of VMM formulations for LCD 3D printing, offering new possibilities for high‐performance ceramic applications in aerospace, automotive, and biomedical industries.

## Graphical abstract

This study highlights the potential of silicon oxycarbide‐based formulations for producing polymer‐derived ceramics with superior properties via LCD 3D printing. VMM‐based ceramics demonstrate a remarkable 50‐fold increase in compressive strength (up to 17 MPa) compared to SPR‐684, along with exceptional high‐temperature survivability (up to 1500 °C) and porosity‐free structures, making them ideal for demanding aerospace and industrial applications.

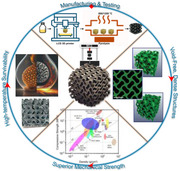

## 1 Introduction

Polymer‐derived ceramics (PDCs) are a class of ceramics synthesized through the thermal decomposition of polymeric precursors. Unlike conventional powder‐based ceramics that require powder processing and sintering, PDCs utilize the molecular architecture of polymers as the starting material. These polymers are converted into ceramics through heat treatment at temperatures typically ranging from 600 to 1200 °C.^[^ ^1^, ^2^ ^]^ Traditionally, PDC components are fabricated by shaping preceramic polymers (PCPs) into the desired form using pressing, extrusion, or injection molding, followed by pyrolysis in an inert atmosphere, which transforms the organic components into the final inorganic ceramic. Due to their excellent thermal stability, oxidation resistance, and mechanical properties, PDCs have applications across microelectronics, biomedicine, aerospace, and optics.^[^ ^3^ ^]^ However, imperfections such as porosity and inhomogeneity significantly impact their strength and reliability. Unlike metals, ceramics are brittle and highly susceptible to crack propagation.^[^ ^4^, ^5^, ^6^, ^7^, ^8^ ^]^ Addressing these challenges is essential to fully harnessing the unique properties of ceramics.

Additive manufacturing (AM) offers a promising pathway by enabling the fabrication of intricate geometries and fine features that can be converted to dense ceramic, reducing porosity and inhomogeneity challenges in PDCs.^[^ ^9^, ^10^, ^11^ ^]^ Various AM techniques have been explored for the 3D printing of PDCs, including direct ink writing, stereolithography (SLA), digital light processing (DLP), two‐photon polymerization (TPP), binder jetting, fused deposition modeling, inkjet printing, and selective laser curing.^[^ ^12^, ^13^, ^14^, ^15^, ^16^, ^17^ ^]^ These advanced techniques enable the fabrication of highly complex and customized structures, unlocking new possibilities for PDC applications in fields requiring design freedom, rapid prototyping, and material precision.

Among AM technologies, SLA is widely recognized for its ability to produce highly detailed and precise structures.^[^ ^18^ ^]^ This makes it ideal for fabricating intricate features with smooth surface finishes.^[^ ^19^ ^]^ Additionally, SLA supports a broad range of compatible resins, offering material properties such as biocompatibility, high‐temperature resistance, and flexibility, making it suitable for diverse applications.^[^ ^20^ ^]^ A key variation of SLA is masked stereolithography (MSLA)‐a vat photopolymerization (VPP) process that selectively cures liquid resin layer‐by‐layer using a masked light source, such as a liquid crystal display (LCD) screen.^[^ ^21^ ^]^ LCD‐based printing offers several advantages over traditional SLA and DLP, including increased printing speed and cost efficiency. Unlike SLA, which employs a point‐by‐point laser curing process, LCD printing cures entire layers simultaneously, significantly improving throughput. Commercial LCD 3D printers range from ≈$150 to $800, with resolutions up to 12K (>58 million pixels) and pixel sizes between 18–50 µm, surpassing the pixel density and affordability of DLP systems.^[^ ^22^ ^]^ While the availability of specialized resins for LCD printing is still growing, its scalability and cost‐effectiveness make it an attractive option for ceramic AM applications. Advancements in 3D printing of recyclable waste‐based ceramics, utilizing slurry systems and organic‐inorganic resins from industrial by‐products, demonstrate potential for sustainable manufacturing.^[^ ^23^, ^24^ ^]^ Integrating these innovations with the scalability and precision of LCD printing could further enhance the environmental impact and expand the applications of AM technologies^[^ ^9^, ^25^ ^]^.

Recent studies on PDC lattices have demonstrated that their mechanical properties depend significantly on precursor chemistry, pyrolysis conditions, and lattice design.^[^ ^26^ ^]^ Compression tests of additively manufactured SiOC‐based lattices have shown that well‐optimized structures can achieve strength‐to‐weight ratios comparable to metal foams and advanced composites.^[^ ^27^, ^28^ ^]^ For example, SiOC lattices fabricated using stereolithography have exhibited compressive strengths ranging from 1.5 to 19 MPa, with variations influenced by relative density and pyrolysis temperature.^[^ ^27^ ^]^ Similarly, LCD‐printed SiOC scaffolds have demonstrated high compressive strengths while maintaining controlled porosity.^[^ ^28^ ^]^ Other advancements in ceramic AM include two‐photon polymerization direct laser writing (TPP‐DLW) and laser lithography, which enable the production of ultrastrong, ductile SiOC micro‐ and nanostructures with exceptional mechanical properties, including strengths exceeding 7 GPa and plastic deformation at the microscale.^[^ ^29^, ^30^ ^]^ While these methods achieve outstanding resolution and surface quality, making them ideal for applications requiring nanoscale precision, their scalability remains limited. Specifically, producing structures thicker than 2 mm often leads to increased internal porosity and structural inconsistencies.^[^ ^31^ ^]^ This highlights the need for complementary approaches, such as LCD‐based AM, which is better suited for fabricating larger, high‐performance ceramic components.

Recent efforts in 3D printing of ceramics have emphasized the development of formulations to improve the printability, mechanical properties, and thermal stability of PDCs.^[^ ^32^, ^33^, ^34^, ^35^, ^36^ ^]^ Among these precursors, SPR‐684, a commercially available SiOC resin from the Polyramic family (Starfire Systems, Inc., Schenectady, NY, USA), has been widely studied in ceramic AM.^[^ ^37^ ^]^ However, like many PCP formulations, SPR‐684 faces challenges such as slow curing kinetics, high viscosity, and susceptibility to porosity and void formation during pyrolysis, which can compromise the structural and thermal integrity of the final ceramics.^[^ ^38^, ^39^ ^]^ To address these limitations,^[^ ^40^, ^41^ ^]^ this study explores an alternative Si‐O‐C precursor, poly(vinylmethoxysiloxane) (VMM‐010), optimized for compatibility with LCD printing. Both VMM‐ and SPR‐based formulations represent recent advances in 3D‐printed ceramics, and this study systematically compares their mechanical and thermal performance, providing a comprehensive evaluation of these formulations for PDCs. The results highlight the superior properties of VMM‐based formulations, including faster curing speed, lower viscosity, enhanced structural integrity, and high‐temperature survivability, enabling the production of dense, porosity‐free ceramics. The introduction of graded structures optimized for void minimization and high‐temperature survivability up to 1500 °C further positions VMM‐based ceramics for demanding applications in aerospace and defense. Leveraging affordable LCD 3D printing technology, this study demonstrates the fabrication of high‐performance PDCs using a VMM‐based resin formulation, with compatibility even for low‐cost (<$500) 3D printers due to the low viscosity and optimized reaction kinetics of VMM‐010. Intricate ceramic structures, such as Schwarz Triply Periodic Minimal Surface (TPMS) geometries, are produced with high precision, low porosity, and favorable properties as evaluated through X‐ray micro‐tomography, energy‐dispersive X‐ray spectroscopy (EDX), density measurements, compression testing, and high‐temperature survivability assessments. Additionally, the influence of critical factors such as pyrolysis temperature (800 °C vs 1200 °C) and feature thickness on ceramic properties is thoroughly explored. Based on these assessments, the VMM‐based formulation shows promise for producing robust, high‐strength SiOC ceramics capable of withstanding mechanical stress and high‐temperature oxidation, making them promising for demanding applications in aerospace, automotive, and other high‐performance fields.

## 2 Materials and Methods

### 2.1 Lattice Design

**Figure 1:**
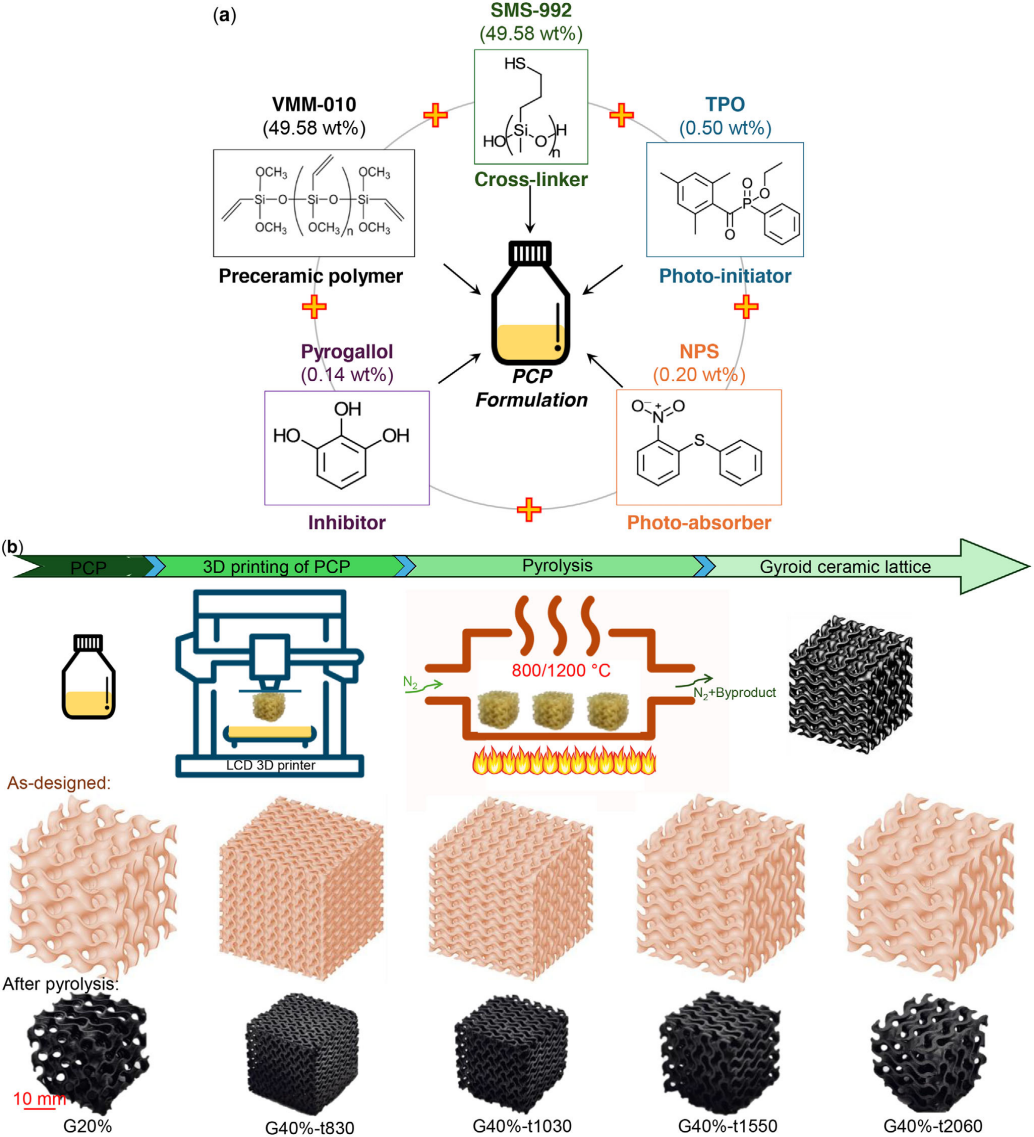
a) Components of the UV‐curable VMM‐based formulation. b) Process flow for producing PDCs from PCPs, including steps for 3D printing, pyrolysis, and mechanical testing. Optical images of Schwarz G structures are shown at different stages: as‐designed (top) and post‐pyrolysis (bottom) for structures with 20% and 40% RDs.

TPMS structures are a distinct class of 3D cellular architectures generated through mathematical techniques.^[^ ^42^, ^43^ ^]^ In our recent study,^[^ ^37^ ^]^ we investigated three types of sheet‐based Schwarz Structures: Primitive (P), Diamond (D), and Gyroid (G). In this work, we focus specifically on the G structure (30 × 30 × 30 mm^3^) with two nominal relative densities (20% and 40%, where relative density (RD) = *ρ/ρ_s_*, with *ρ* as the density of the structure and *ρ_s_* as the solid material density), as shown in **Figure** **1**b. The Gyroid was selected due to its advantageous mechanical characteristics, including an optimal balance of strength and energy absorption, and its ability to minimize stress concentrations due to its smooth, continuous surface and zero mean curvature. This unique geometry allows for efficient load distribution, enhancing structural resilience and stability under compression, making the Gyroid particularly suitable for high‐performance applications requiring robust strength‐to‐weight ratios. For the Schwarz G lattice with 40% RD, we adjusted the design to investigate the effect of maximum feature thickness on printability and mechanical properties. By reducing the maximum feature thickness and increasing the number of unit cells, we maintained a constant 40% RD across different designs. Specifically, we developed four distinct G lattices, each with the same 40% RD but varying maximum feature thicknesses. This approach allows us to analyze how feature thickness influences both printability and mechanical performance. **Table** **1** details the configurations used in this study. For instance, the designation G40‐t2060 refers to a Schwarz G lattice with 40% RD and a maximum feature thickness of 2060 µm.

**Table 1:** Configurations of 3D‐printed Schwarz G lattice samples with varying PDC formulations, RDs, unit cell sizes, maximum feature thicknesses, and total unit cell numbers used in this study.

Sample Name	PDC Formulation	Relative Density (RD)	Unit Cell Size (mm^3^)	Max. Feature Thickness (µm)	Unit Cell Number (Total)
G40‐t2060‐SPR	SPR‐684	G40%	10 × 10 × 10	2060	3 × 3 × 3 (27)
G20‐t1030	VMM‐010	G20%	10 × 10× 10	1030	3 × 3 × 3 (27)
G40‐t2060	VMM‐010	G40%	10 × 10 × 10	2060	3 × 3 × 3 (27)
G40‐t1550	VMM‐010	G40%	7.5 × 7.5 × 7.5	1550	4 × 4 × 4 (64)
G40‐t1030	VMM‐010	G40%	5 × 5 × 5	1030	6 × 6 × 6 (216)
G40‐t830	VMM‐010	G40%	4 × 4 × 4	830	7.5 × 7.5 × 7.5 (422)

### 2.2 Resin Formulation for 3D Printing

The formulation (see Figure 1a) for 3D‐printable PCPs includes a ceramic precursor, photoinitiator, cross‐linker, inhibitor, and photoabsorber, creating a resin compatible with 3D printing and capable of achieving precise ceramic geometry after printing and pyrolysis. Typically, small amounts (≈0.5% or less) of photoinitiators, absorbers, and inhibitors are used, while the cross‐linker, critical for rapid and controlled gelling, comprises ≈50 wt.% of the formulation. In this study, the formulation is based on a Si‐O‐C precursor, poly(vinylmethoxysiloxane) (GEL‐VMM‐010, CAS No. 131298‐48‐1, GELEST, USA), which contains vinyl and methoxy groups. To achieve the rate and control necessary for 3D printing, a polythiol cross‐linker, poly(mercaptopropylmethylsiloxane) (GEL‐SMS‐992, CAS No. 102783‐03‐9, GELEST, USA), was selected. These Si‐O‐C precursors were selected for their stability to oxygen and moisture, simplifying handling and enabling the use of the resin in commercial 3D printers without modifications. To control voxel size and prevent overexposure, 2‐nitrophenyl phenyl sulfide (Tokyo Chemical Industry Co., Japan, CAS No. 4171‐83‐9) was chosen as a photoabsorber. TPO (Diphenyl(2,4,6‐trimethylbenzoyl)phosphine oxide; Sigma–Aldrich, Germany) was used as the photoinitiator to initiate polymerization, owing to its high light absorption at the wavelength of the printer. Pyrogallol (Molekula Americas LLC, USA, CAS No. 87‐66‐1) served as an inhibitor, effectively stabilizing thiol‐ene formulations. All components were mixed in a Thinky ARE‐310 planetary mixer (Thinky Inc., CA, USA) for 10 min at 2000 rpm, with a 30‐s defoaming at 2200 rpm before each print. A second formulation based on another polysiloxane resin (SPR 684, Starfire Systems, USA), as reported in our previous work,^[^ ^37^ ^]^ was employed for comparison. The contents of formulations are itemized in the Supporting Information (see Table S1,S2, Supporting Information).

### 2.3 Rheological Characterization

To evaluate the curing parameters of our PCP resin, parallel plate photorheology measurements were conducted using a Discovery HR‐2 Rheometer, equipped with an Omnicure S2000 Spot UV Curing System (200‐Watt high‐pressure mercury vapor short arc lamp), a light guide, and a Thorlabs optical bandpass filter (Model FBH405, CWL 405 nm, FWHM 10, 25 mm diameter). The light intensity was calibrated using a Thorlabs PM100D Compact Power and Energy Meter Console with a Standard Photodiode Power Sensor (Model S121C, 400–1100 nm, 500 mW). Measurements were taken with 20 mm diameter acrylic plate and a 100 µm gap to replicate the 3D‐printing conditions. The instrument operated in oscillation mode, with a rotational frequency of 0.5 Hz and strain set at 0.3%. Viscosity measurements were obtained during these experiments to compare the flow behavior of VMM‐010 and SPR‐684 formulations. Each experiment began with a 60‐s temperature soak to allow samples to acclimate to room temperature. A 30‐s pre‐exposure measurement confirmed temperature stabilization through steady storage and loss modulus readings. Following this delay, the shutter was opened, exposing the sample to light for the remainder of the test.

### 2.4 3D Printing

The 3D printing process utilized an LCD 3D printer (ELEGOO Saturn 3 12K, China) equipped with a 10‐inch 12K Mono LCD. Each layer was 50 µm thick and cured using a LED light source (405 nm wavelength, 2.2 mW cm^−^^2^ at the resin surface). The corresponding exposure dose ranged from 60 to 120 mJ cm^−^^2^, depending on layer geometry and printing parameters. This LCD 3D printer provides a pixel size of 19 µm × 24 µm in the XY plane; however, due to practical factors such as resin viscosity and curing dynamics, the smallest features reliably fabricated without defects were ≈57 µm (3 pixels). It should be noted that this measurement is before pyrolysis and, due to material shrinkage during pyrolysis, the final smallest feature size achieved is even smaller. For the *Z*‐axis, the parameters and photoabsorber concentrations were optimized for a layer thickness of 50 µm, which is slightly lower than the penetration depth of light at the given exposure time. Improved Z resolution could potentially be achieved by increasing the photoabsorber concentration, which would reduce the penetration depth and allow for thinner layer thicknesses.

Printing speed was determined by the layer curing time and the mechanical movement of the build plate. With a standard exposure time of 50 s per layer and a lifting speed of 20 mm s^−1^, the printer achieved a vertical throughput of ≈3 mm h^−1^. While typical for high‐resolution LCD printing, throughput can be optimized with faster‐curing formulations or adjustments to layer thickness for application‐specific requirements. Before printing, the platform was cleaned with isopropyl alcohol (IPA). After the printing cycle, fabricated parts were cautiously removed using a box cutter and washed with IPA to remove uncured resin. Finally, prints were post‐cured using a UV curing chamber (Model DR‐301C, 405 nm Asiga, Australia). **Table** **2** provides the 3D printing parameters for the LCD printer.

**Table 2:** ELEGOO Saturn 3 12K printing parameters.

Printing Parameters		Peeling Parameters	
Layer height	0.05 mm	Rest time before lift	1 s
Exposure time	50 s	Rest time after retract	3 s
Bottom exposure time	70 s	Lifting speed	20 mm s^−1^
Waiting mode during printing	Resting time	Retract speed	150 mm s^−1^

### 2.5 Pyrolysis

Pyrolysis was conducted under nitrogen purge (50 SCFH) in a box furnace, with samples elevated off the base of the furnace on alumina tubes and plates to ensure uniform temperature distribution. The heating ramp rate was set to 1 °C min up to 800 and 1200 °C, with holds of 4 h at 350 °C for maximum polymerization (pre‐pyrolysis) and 4 h at 800 °C (or 1200 °C) for pyrolysis and ceramization. Pyrolysis progress was further assessed using thermogravimetric analysis (TGA) coupled with Fourier‐transform infrared (FTIR) spectroscopy (Netzsch STG 449 F1 at 10 °C min^−1^ heating rate under argon, with an in‐line Bruker Tensor 27 FTIR spectrometer).

### 2.6 Mechanical Testing

Uniaxial compression tests were conducted using a universal testing machine (Instron 5900R, Instron, USA) equipped with a 5 kN load cell. The test rate and other relevant parameters were selected based on the guidelines of ASTM C1424‐15 to ensure consistent quasistatic compression conditions at room temperature. Specifically, the machine operated at a crosshead speed of 0.5 mm min^−1^. While the sample size and geometry differed from the standard due to the unique nature of the 3D‐printed structures, the selected parameters allowed for reliable comparison across different sample types and densities. A camera recorded the tests, enabling detailed analysis of load‐displacement data and visual observations of deformation and failure mechanisms. Three samples were tested for each type and density of the Schwarz structures to ensure reproducibility of the results.

### 2.7 PDC Characterization: Shrinkage, Voids, and Density

Shrinkage was assessed by measuring the outer dimensions of the lattice parts before and after pyrolysis and using these average linear dimensions to calculate linear shrinkage. Ceramic density was measured with an AccuPyc 1330 pycnometer (Micromeritics Instrument Corp., GA, USA). X‐ray micro‐computed tomography (Micro‐CT) imaging was performed using a ZEISS Xradia 520 Versa X‐ray microscope (Pleasanton, CA, USA) to study the 3D distribution of cracks and voids as well as other features within the 3D‐printed ceramics. The experimental setup included an X‐ray source, a sample, and a detector, with source‐sample and sample‐detector distances adjusted for maximum magnification. The source‐sample distance was set at 110 mm and the sample‐detector at 65 mm, with exposure times of 3 s for green bodies and 1.5 s for ceramics to achieve optimal signal‐to‐noise levels (intensities >5000 gray value in low transmission regions). The tungsten source emitted a continuous X‐ray spectrum, and transmission through green bodies was optimized by narrowing the X‐ray bandwidth with low‐energy filters (520 Versa LE3 filter for green bodies and LE5 for ceramics), adjusting the applied voltage. Only the LE2 filter was used for PDCs. Projection scans were acquired at 60 kV and 5 W, resulting in 35–40% transmission, with a total of 1601 2D scans captured over a 360° rotation. The tomography datasets were reconstructed using ZEISS Reconstructor software and analyzed with Dragonfly (premium version) software for 3D segmentation and assessment.

### 2.8 SEM‐EDX Compositional Analysis

Three ceramic samples of each type were embedded in resin and polished to provide a smooth surface for SEM‐EDX analysis. Multiple regions of each sample were examined to obtain representative compositional data. The analysis was conducted using a Hitachi SU3500 scanning electron microscope equipped with an Oxford Instruments X‐act EDX detector.

### 2.9 Data Analysis

The results in this manuscript are presented as the mean ± standard deviation over three independent experiments (*n* = 5), unless specified otherwise in the figure captions.

## 3 Results and Discussion

### 3.1 Polymer Characterization

**Figure 2:**
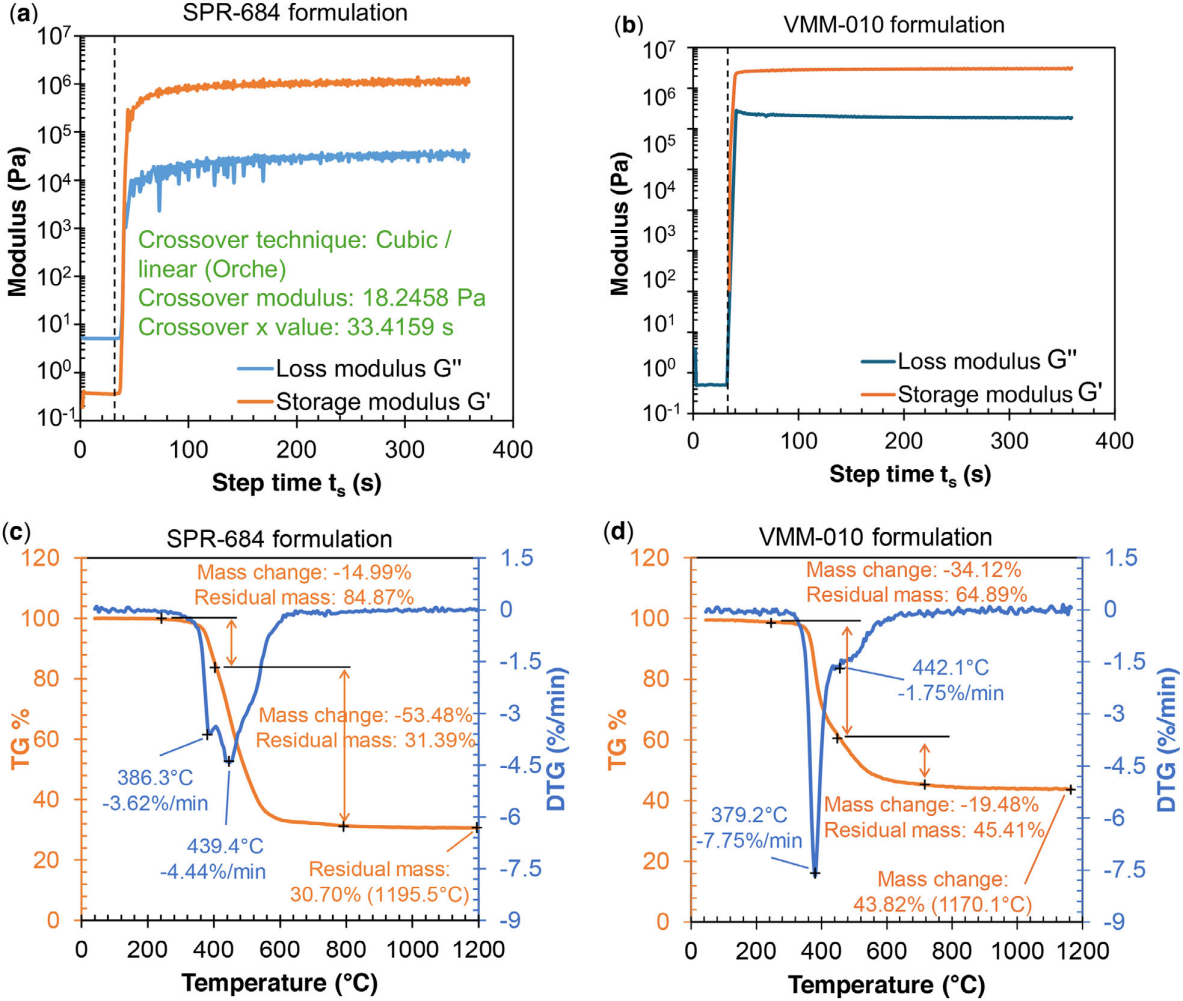
Comparison of curing and pyrolysis behavior between SPR‐684 and VMM‐010 based formulations. a,b) Photorheology measurements under 405 nm UV light at 20 mW cm^−^^2^, with SPR‐684 achieving modulus crossover at ≈3 s. c,d) Thermogravimetric analysis (TGA) of both formulations, shows primary mass loss peaks ≈445 °C, with minimal mass loss beyond 800 °C, indicating near‐complete pyrolysis.

**Figure** **2** compares the curing and pyrolysis behavior of two formulations: SPR‐684 and VMM‐010. Photorheology measurements for both formulations reveal their respective curing kinetics under UV light exposure. The VMM formulation shows a steeper slow and higher maximum storage modulus. The modulus crossover of ≈3 s of exposure, indicates rapid curing compatible with 3D printing methods. In contrast, the SPR‐684 formulation exhibits a longer time to modulus crossover, indicating a slower curing rate and lower maximum storage modulus.

The VMM formulation was also measured to have lower viscosity (25.5 ± 0.5 cP) in comparison to the SPR formulation (3000.5 ± 0.5 cP). This facilitates washing of printed samples and reduces the likelihood of uncured resin being trapped within complex geometries, which is a common issue with higher‐viscosity resins like the SPR formulation. Additionally, lower viscosity reduces the separation force during the layer‐by‐layer printing process, allowing for increased separation speed and potentially reducing overall printing time. Furthermore, at the green stage, VMM‐based prints are more rigid compared to the softer SPR‐684 prints. This improved rigidity enhances the stability of the parts during printing and minimizes the occurrence of defects. The reduced resin entrapment in VMM‐based prints contributes to their superior structural integrity and ensures a more uniform polymer‐to‐ceramic transformation during pyrolysis. Additionally, the lower viscosity of VMM formulations likely enhances the crosslinking rate, supporting the observed faster modulus crossover in photorheology measurements.

TGA results (see Figure 2c,d) illustrate the mass loss profiles of both formulations during pyrolysis. The VMM‐based formulation shows a primary mass loss peak near 445 °C, while SPR‐684 has a similar peak at ≈445.4 °C. Beyond this point, further mass loss in both formulations is minimal, indicating near‐complete pyrolysis by 800 °C. The mass loss curves demonstrate that both formulations follow similar thermal decomposition paths, though the VMM‐based formulation provides higher ceramic yield (higher residual mass) after pyrolysis.

### 3.2 PDC Composition, Shrinkage, and Density

**Table** **3** describes the composition of ceramics derived from the SPR‐684 and VMM‐010 formulations. Upon pyrolysis, polysiloxane‐based ceramic precursors result in an amorphous SiOC matrix with residual turbostatic carbon domains.^[^ ^2^ ^]^ This amorphous structure is stable up to pyrolysis temperatures of 1450 °C.^[^ ^44^ ^]^ The composition of the final ceramic is influenced by the chemical structure of the preceramic polymer (e.g., Si/O ratio), the pyrolysis atmosphere, and the temperature.^[^ ^45^, ^46^ ^]^ Ceramics derived from the SPR formulation exhibited higher carbon content, which may be partially attributed to the increased carbon present in aromatic hydrocarbon rings in the precursor polymer.^[^ ^47^, ^48^ ^]^ While TGA‐FTIR analyses indicate the release of aromatic compounds during pyrolysis of the SPR formulation, the exact contribution of this carbon to both the lower ceramic yield and the higher free carbon content in the final PDC remains unclear. In contrast, the VMM formulation, which lacks aromatic groups, produced ceramics with higher ceramic yield and lower free carbon content at the same pyrolysis temperature. VMM‐based samples pyrolyzed to higher temperatures (1200 °C) showed even lower carbon content. During polymer‐to‐ceramic transformation, occurring between 600 and 800 °C, free carbon precipitates within the ceramic matrix. At higher pyrolysis temperatures, sp^3^‐hybridized carbon transitions to sp^2^‐hybridized carbon, facilitating the restructuring of the amorphous ceramic network.^[^ ^49^, ^50^ ^]^ A small amount of sulfur detected in the final ceramic is attributed to the thiol cross‐linker used during photocuring. The mechanical properties of the resulting ceramics are closely tied to their composition. Notably, the reduced free carbon content in VMM‐010‐derived ceramics correlates with improved stiffness and hardness, as free carbon is known to inversely affect Young's modulus and hardness in SiOC ceramics.^[^ ^51^ ^]^ These compositional differences underscore the advantages of the VMM‐010 formulation in producing ceramics with superior mechanical performance and structural integrity.

**Table 3:** EDX composition analysis of ceramics derived from SPR‐684 and VMM‐010 formulations and pyrolysis temperatures, highlighting variations in Si, C, O, and S content and the resulting stoichiometry of the silicon‐oxycarbide phases.

PCP formulation	Pyrolysis Temperature (°C)	Atomic wt.%				Composition
		Si	C	O	S	
SPR‐684	800	14.38	53.30	32.01	0.19	SiC_8.64_O_3.897_S_0.01_
VMM‐010	800	17.62	39.99	41.33	0.76	SiC_5.29_O_4.10_S_0.03_
VMM‐010	1200	25.16	31.08	42.53	1.23	SiC_2.88_O_2.95_S_0.04_

**Figure 3:**
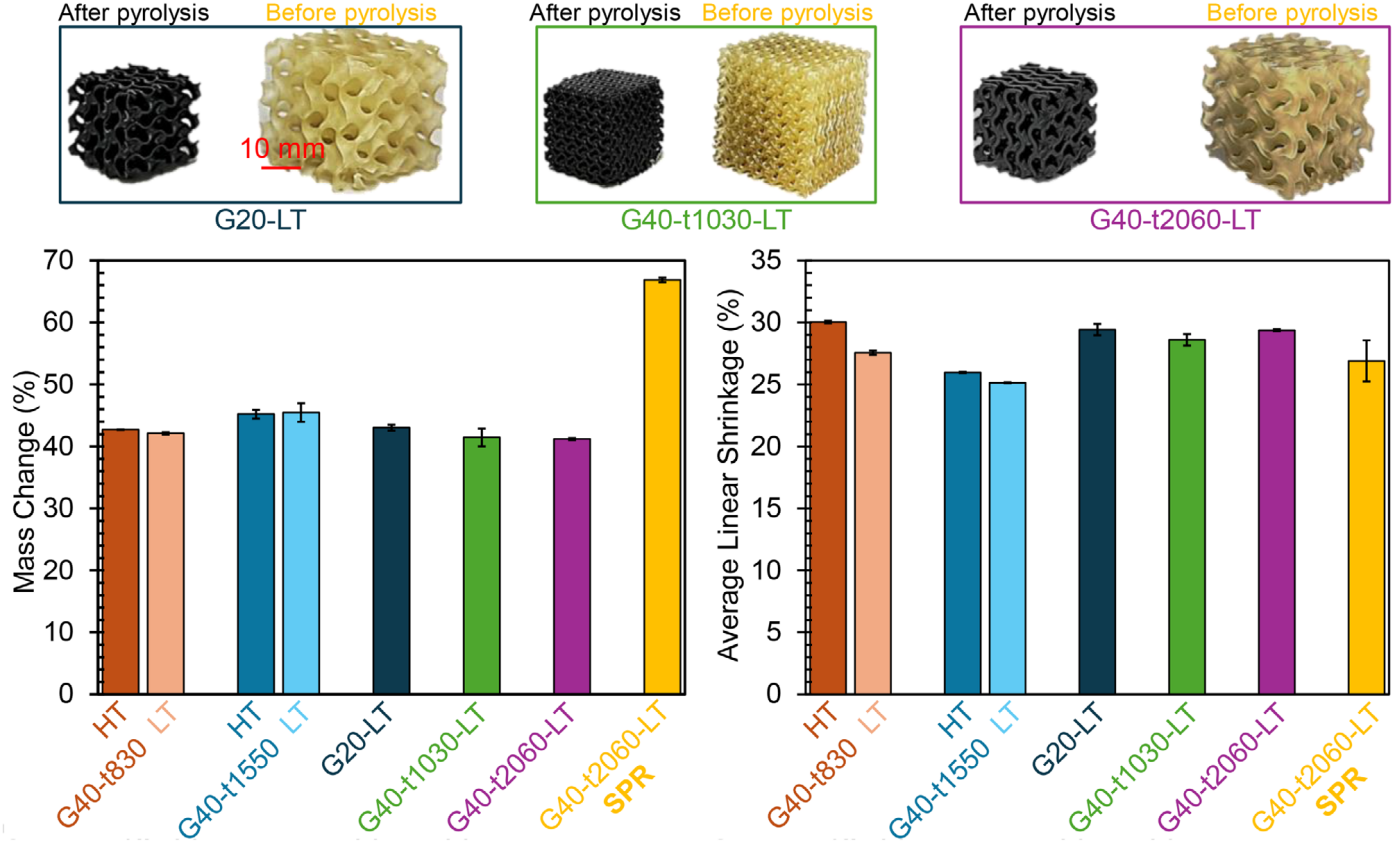
a) Mass change, and b) Average linear shrinkage of 3D‐printed Schwarz G ceramic structures after pyrolysis, illustrating the effects of pyrolysis on mass retention and dimensional stability in the printed samples.

**Figure** **3**a,b depict the mass and linear shrinkage of Schwarz G lattice samples with 40% RD following pyrolysis. Figure 3a illustrates mass shrinkage, showing that different lattice structures with varying feature thicknesses, but the same RD (40%), exhibit nearly identical mass change percentages after pyrolysis. This consistency suggests uniform material conversion across the samples, regardless of structural thickness. Figure 3b displays linear shrinkage, calculated as the average shrinkage along three orthogonal directions. Similar to mass shrinkage, the linear shrinkage values are closely aligned across the samples with 40% RD, indicating reliable dimensional stability during the pyrolysis process.

Helium pycnometry measurements were conducted to verify the ceramic densities of the VMM‐based lattice samples. The results showed ceramic densities of 1.828 ± 0.005 g cm^−^^3^ for the G20 structure, 1.819 ± 0.003 g cm^−^^3^ for the G40 structure with varied thickness, and 1.834 ± 0.001 g cm^−^^3^ for the G40 structure with modified unit size. These values fall within the expected density range for SiOC ceramics (typically 1.50–1.95 g cm^−^^3^^[^ ^10^ ^]^), and are compared to similar lattices produced by the SPR‐formulation (1.56–1.63 g cm^−^^3^^[^ ^37^ ^]^), indicating that the VMM‐based formulation results in dense ceramic structures with minimal void content. The consistency across different configurations suggests that the VMM‐based PDC maintains uniform material properties within the range of relative density or structural adjustments in thickness and unit size explored here.

### 3.3 Micro‐CT

**Figure 4:**
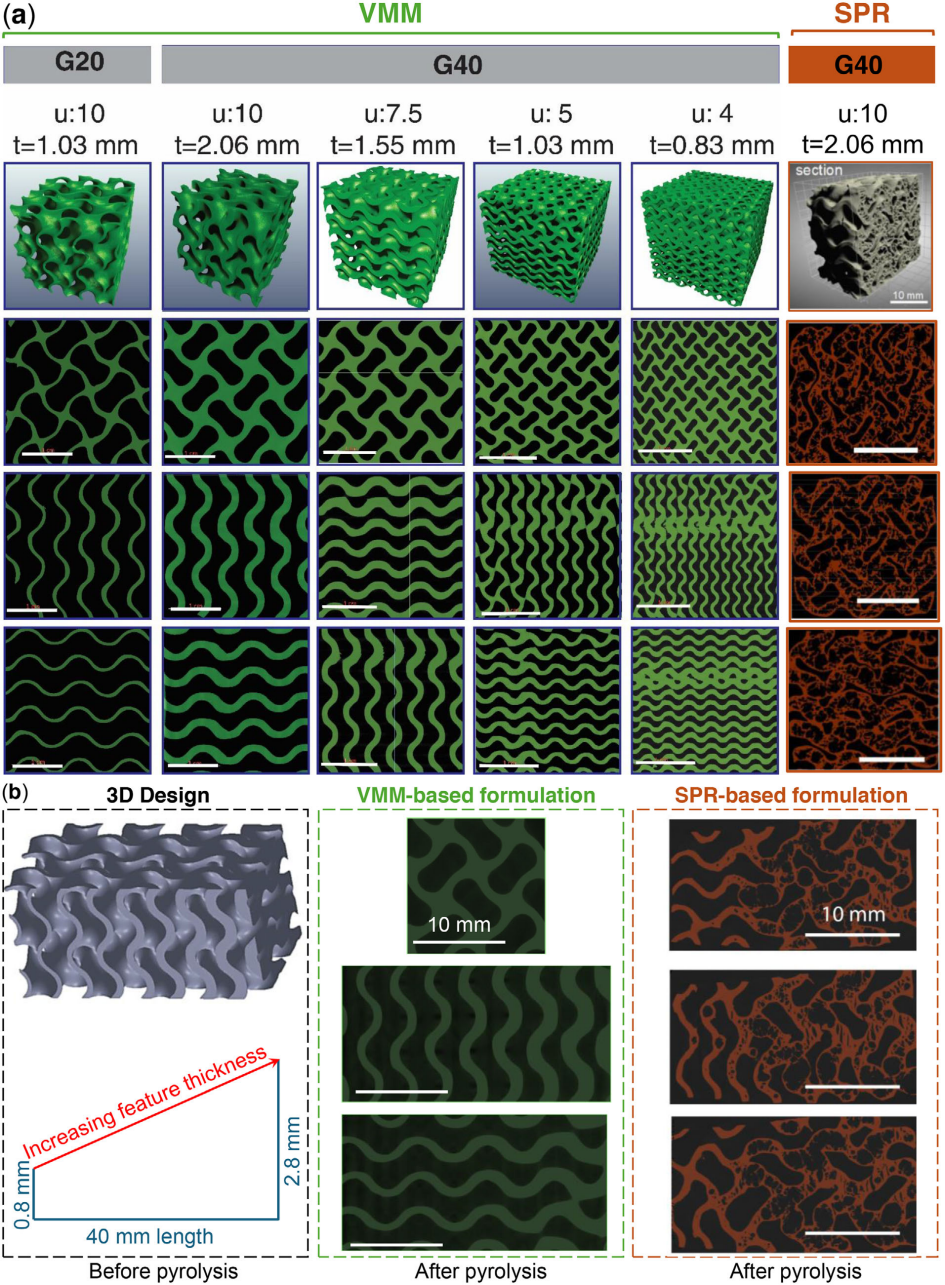
a) Micro‐CT scans of 3D‐printed gyroid structures post‐pyrolysis. Left: Cross‐sections of a gyroid structure with a RD of 20%, showing the structural uniformity of the VMM‐based formulation. Right: Cross‐sections of G40 structures with varying feature thicknesses and unit cell sizes, including both VMM and SPR‐based formulations. The SPR sample reveals greater distortion and porosity compared to the VMM counterpart, particularly in smaller unit cells. Reducing unit cell size introduces some distortion, as observed in the smallest unit cell (4 mm cube) of the G40 structures. Achieving a balance between unit cell size and feature thickness is essential for maintaining target relative density. Scale bar = 10 mm; “u” denotes unit cell size in mm, and “t” denotes maximum feature thickness. b) Graded gyroid structure based on VMM and SPR formulations with varying thicknesses ranging from 0.8 to 2.8 mm along a 40 mm length, designed to identify the optimal thickness for void‐free fabrication. The structure was 3D‐printed and evaluated through CT scans post‐pyrolysis to analyze its microstructural integrity and performance.

The CT scans provide compelling evidence of the effectiveness of the VMM‐based formulation in yielding dense ceramic material within gyroid structures (see **Figure** **4**a). These scans confirm the structural fidelity of both G20 and G40 samples, with 20% and 40% RDs, respectively. Notably, the VMM‐based ceramics preserved the intricate gyroid lattice architecture across various thicknesses and unit cell sizes, demonstrating the robustness and versatility of the formulation.

A critical observation is the absence of porosity within the micro‐CT images of pyrolyzed VMM‐based ceramics, even at the higher RDs and feature thicknesses. This represents a substantial improvement compared to SPR‐based ceramics, which exhibited significant void formation and structural distortion in G40 lattices post‐pyrolysis. Previous studies^[^ ^37^ ^]^ attributed such voids to inefficient gas escape during pyrolysis, particularly in thicker lattices, leading to gas trapping and differential shrinkage. SPR‐based G40 samples showed extensive void formation and distortion, often resulting in inner structures coming into contact.

To further investigate the influence of feature thickness, graded gyroid structures with varying thicknesses (0.8 to 2.8 mm) along a 40 mm length were fabricated using both formulations (see Figure 4b). Post‐pyrolysis CT scans revealed that SPR‐based ceramics began developing voids at thicknesses exceeding 1.5–2 mm due to inefficient gas release. In contrast, VMM‐based ceramics remained void‐free across all thicknesses, including the thickest sections. These results emphasize the superior structural consistency and robustness achieved with the VMM formulation.

While the residual mass differences observed in Figure 2c,d primarily reflect the total decomposition and by‐product release during pyrolysis, they do not capture the spatial dynamics of gas escape, which is critical to void formation. The void‐free nature of VMM‐based ceramics, even in thick sections (see Figure 4b), can be attributed to several interrelated factors. Optimized curing of the VMM‐based formulation and higher ceramic yield may help ensure a consistent and controlled polymer‐to‐ceramic transformation, which facilitates efficient gas escape. Although the low viscosity of the VMM‐based formulation inherently supports easier cleaning; the cleaning protocols were identical for both VMM‐ and SPR‐based samples. Pre‐pyrolysis masses of 7.37 and 7.36 g for the VMM‐based and SPR‐based polymer lattices, respectively, confirm that the printed samples had comparable geometries and were equally cleaned of uncured resin prior to pyrolysis. This indicates that the observed differences in void formation arise from material‐specific advantages of the VMM formulation rather than variations in cleaning or initial sample quality.

While the exact mechanisms of gas management remain speculative, the micro‐CT results of the graded structures (see Figure 4b) serve as the definitive evidence of the superior, nearly void‐free (within the resolution of the micro‐CT) nature of the VMM‐based ceramic. Additionally, the nearly identical pre‐pyrolysis masses indicate that the observed differences are not due to variations in green part geometry or cleaning efficacy but are a direct consequence of the properties of VMM formulation. These combined factors enable the production of dense, structurally consistent ceramics suitable for high‐performance applications where reliability and precision are paramount. Such applications include aerospace, automotive, and biomedical industries, where porosity‐free structures are essential for durability and functional integrity under extreme conditions.

### 3.4 Lattice Performance in Compression

**Figure 5:**
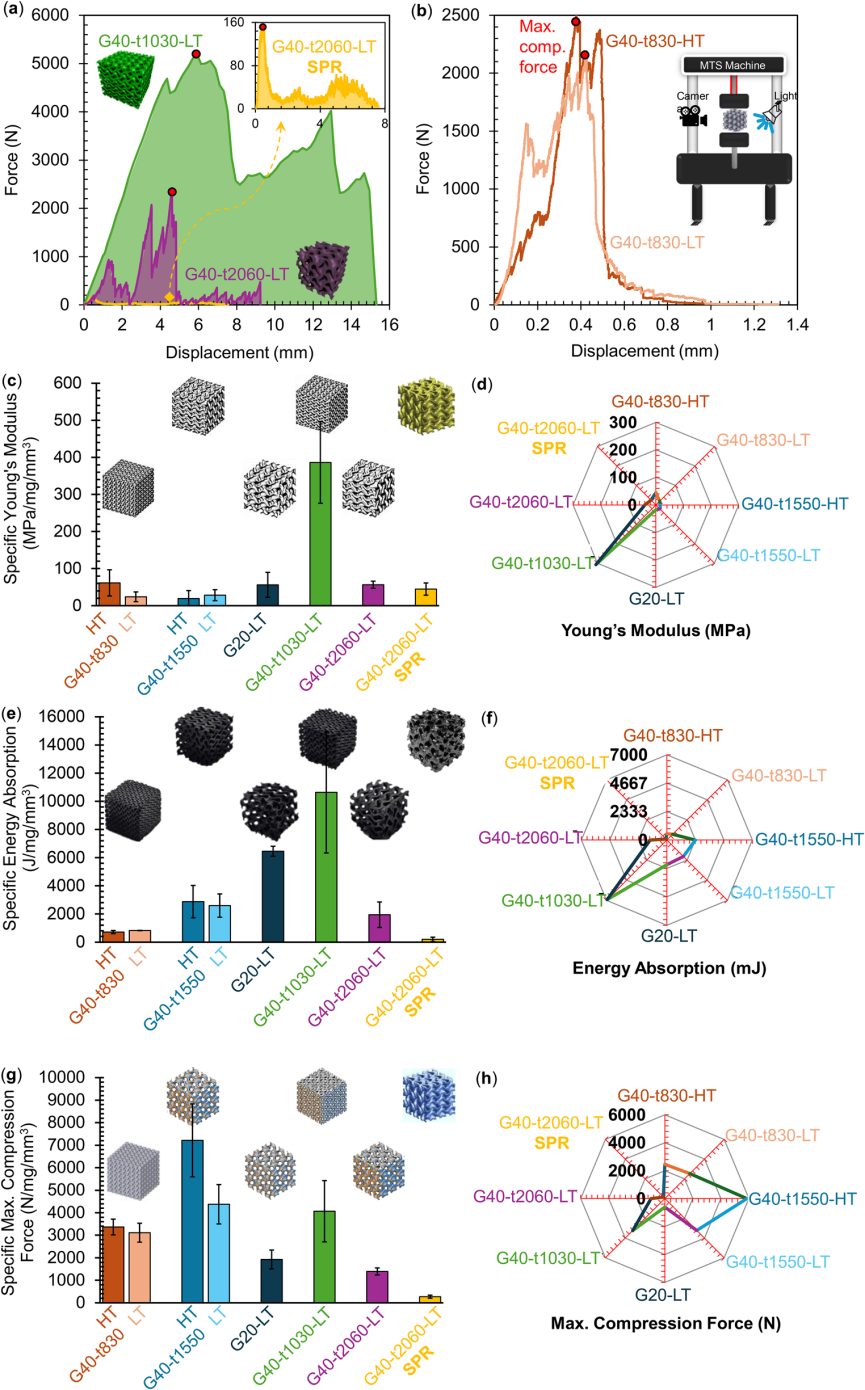
Uniaxial compression test results for Schwarz G lattice samples with varied feature thicknesses, formulations, and pyrolysis conditions. a) Force‐displacement curves comparing VMM‐ and SPR‐based formulations under LT pyrolysis. b) Force‐displacement curves for G40‐t1030 samples with LT and HT pyrolysis. c) Specific Young's modulus, showing rigidity per unit mass. d) Absolute Young's modulus, reflecting stiffness. e) Specific energy absorption, relevant for impact resistance. f) Total energy absorption before failure. g) Specific maximum compression force per unit mass. h) Maximum compression force, indicating absolute strength.

**Figure** **5** presents the results of uniaxial compression tests on Schwarz G lattice samples, evaluating the effects of material formulation (VMM‐010 vs SPR‐684), feature thickness, and pyrolysis conditions (high‐temperature (HT) at 1200 °C versus low‐temperature (LT) at 800 °C) on mechanical performance. The tested configurations, including 20% and 40% RDs with varying feature thicknesses, were analyzed to determine their strength, stiffness, and energy absorption capabilities. Specific mechanical properties were normalized by density to provide a meaningful comparison of strength and stiffness efficiency relative to mass.

The force‐displacement curves in Figure 5a compare VMM‐based samples (G40‐t1030‐LT and G40‐t2060‐LT) with an SPR‐based counterpart (G40‐t2050‐LT^[^ ^37^ ^]^). VMM‐based samples consistently exhibit higher load‐bearing capacity and structural integrity across different feature thicknesses, demonstrating a denser, more cohesive ceramic matrix capable of withstanding greater compressive loads while maintaining structural integrity. This suggests that the VMM‐based formulation provides superior mechanical resilience, making it well‐suited for applications requiring enhanced durability and load‐bearing performance.

Pyrolysis temperature plays a crucial role in mechanical behavior, as highlighted in Figure 5b, which compares LT‐ and HT‐treated samples of G40‐t1030. HT‐treated samples exhibit higher energy absorption and compressive strength due to increased densification, which strengthens interatomic bonds and reduces porosity. In contrast, LT‐treated samples, while demonstrating greater stiffness, exhibit lower energy absorption, making them less ideal for impact‐resistant applications where controlled energy dissipation is essential.

Stiffness properties are further analyzed in Figure 5c,d, which show specific Young's modulus (normalized by density) and absolute Young's modulus, respectively. VMM‐based formulations demonstrate superior stiffness efficiency, achieving greater rigidity without excessive weight. HT pyrolysis generally enhances stiffness due to densification; however, in some cases, such as the G40‐t1550 sample, LT‐treated structures exhibit comparable or even higher stiffness, suggesting that stiffness optimization depends on both processing conditions and structural geometry.

The energy absorption characteristics, shown in Figure 5e,f, highlight the effect of feature thickness and pyrolysis conditions on impact resilience. LT‐treated samples, particularly G40‐t1030, exhibit the highest specific energy absorption, indicating enhanced flexibility and controlled deformation under load. This suggests that an “optimal feature thickness” exists, where strut dimensions balance flexibility and structural stability to maximize energy dissipation. While HT‐treated samples demonstrate increased rigidity, they tend to absorb less energy due to reduced deformability. This trade‐off between stiffness and flexibility underscores the importance of tuning feature thickness and processing conditions for specific application requirements in energy‐dissipative structures.

The load‐bearing capacity of the samples is analyzed in Figure 5g,h, which presents specific and absolute maximum compression forces. HT‐treated samples with optimized feature thicknesses exhibit the highest compression forces, demonstrating the effectiveness of high‐temperature pyrolysis in achieving fully densified, mechanically robust ceramics. This makes HT‐treated samples particularly suitable for high‐stress structural applications requiring maximum strength and minimal deformation.

Overall, the results in Figure 5 reveal that material formulation, feature thickness, and pyrolysis temperature collectively determine the mechanical properties of ceramic lattices. The interplay between these factors influences the balance between stiffness, strength, and energy absorption, enabling the design of tailored lattice architectures for a range of engineering applications. By leveraging the superior densification and load‐bearing capacity of HT‐treated structures, along with the energy absorption capabilities of LT‐treated configurations, VMM‐based formulations offer a versatile approach to optimizing ceramic lattice performance across diverse functional requirements.

**Figure 6:**
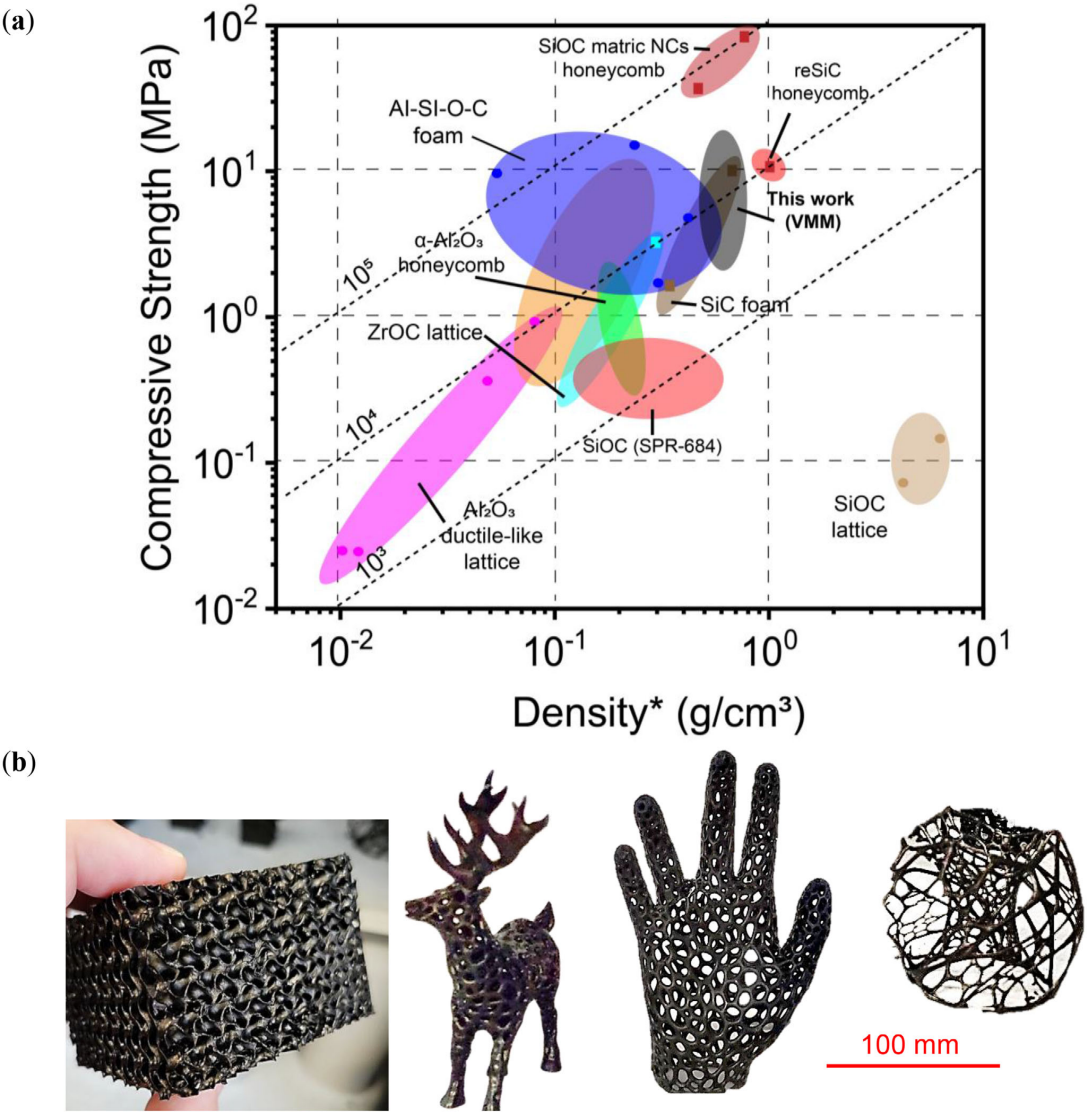
a) Comparative Ashby chart displaying the compressive strength versus lattice density of the 3D‐printed VMM‐based formulation in relation to other PDC and ceramic/composite materials. (Lattice Density* = RD × ceramic density) b) Demonstration of scalability and complexity in 3D printing using VMM‐based formulations: (Left to right) Gyroid structure, reindeer‐shaped lattice, hand‐shaped lattice, and stochastic lattice ball. The structures exhibit high precision and structural fidelity across varying scales and intricate geometries. Scale bar = 100 mm.

**Figure** **6**a compares the compressive properties of VMM‐based lattices relative to other PDCs and similar materials. The VMM‐based SiOC lattices demonstrate a relatively high density (g cm^−^^3^) and a compressive strength range of 1–17 MPa. Positioned near the top of the chart, these lattices exhibit good performance in compressive, which, combined with its moderate density, results in a favorable strength‐to‐weight ratio. Additionally, these lattices are between the 10^2^ and 10^3^ J g^−1^ diagonal lines, highlighting efficient energy absorption and high material efficiency.

As shown in Figure 6a, the compressive strength values demonstrate significant improvements within the context of our study, while also reflecting variations stemming from differences in preceramic formulations, fabrication methods, and structural designs across studies.^[^ ^27^, ^28^, ^52^ ^]^ For example, materials like SiC foam prioritize ultra‐lightweight characteristics at the expense of compressive strength, whereas SiOC ceramics with higher compressive strengths may employ different precursor chemistries or fabrication techniques, leading to variations in density and mechanical performance. In contrast, VMM‐based PDC lattices achieve a combination of substantial compressive strength and moderate density, making them ideal for applications requiring load‐bearing capacity where additional weight is manageable. This versatility positions VMM‐based ceramics as a promising option for structural applications demanding durability and resistance to compressive loads, such as aerospace, automotive, and high‐stress industrial components.

Figure 6b further illustrates the scalability and versatility of the VMM‐based formulation in LCD 3D printing, showcasing a range of complex geometries successfully fabricated. These include a gyroid structure, a reindeer‐shaped model, a hand‐shaped lattice, and a stochastic lattice ball, spanning intricate small components to larger macroscopic features. The ability to print structures with varying complexity and scale underscores the robust printability of the VMM formulation, highlighting its suitability for diverse applications, from structural components to artistic designs. This scalability further emphasizes the formulation's compatibility with different printing resolutions and its ability to maintain structural integrity across geometries of varying complexity. Overall, the performance of the VMM‐based ceramic, as illustrated in Figure 6, underscores its significant potential for advanced engineering applications. With its combination of high compressive strength, moderate weight, and scalability, VMM‐based ceramics offer a robust, load‐bearing solution for high‐performance industrial and engineering roles.

### 3.5 High‐temperature Survivability

**Table** **4** presents the high‐temperature survivability results for 3D‐printed ceramic samples with a 40% RD derived from the two formulations. The SPR‐based sample, pyrolyzed at 800 °C, exhibited a compressive strength of 0.8 MPa but was substantially destroyed after a 2‐h exposure at 1500 °C in air. In contrast, the VMM‐based ceramics demonstrated remarkable high‐temperature resilience. The VMM‐based sample pyrolyzed at 800 °C retained structural integrity with only a 12% weight loss, while the same samples pyrolyzed at 1200 °C exhibited no weight loss, indicating good stability under extreme thermal conditions. This exceptional thermal stability in VMM‐based ceramics can be attributed to increased ceramic densification and robust interatomic bonding achieved during HT pyrolysis at 1200 °C, which imparts greater thermal resilience to the VMM matrix. These structural improvements reduce porosity and enhance the continuity of the ceramic network, allowing the material to withstand thermal stresses without degradation. Such high thermal resilience highlights the potential of VMM‐based ceramics for high‐demand applications, particularly in aerospace and defense, where materials must endure extreme temperatures while maintaining both mechanical and thermal integrity. The ability of VMM‐based formulations to retain strength and stability under these conditions underscores their suitability for advanced engineering applications that require both durability and thermal resistance.

**Table 4:** High‐temperature survivability of 3D‐printed ceramic samples with 40% RD (G40%) using SPR‐684 and VMM‐010 based formulations.

3D printed Ceramic Sample	Pyrolysis Temperature (°C)	Compressive Strength (MPa)	Weight loss (2 h at 1500 °C, Air)	Visual Inspection
G40‐t2060 (SPR‐684)	800	0.8 ± 0.1	Destroyed	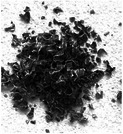
G40‐t2060 (VMM‐010)	800	8.0 ± 0.2	12%	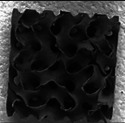
G40‐ t2060 (VMM‐010)	1200	16.6 ± 0.4	0.0%	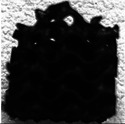

## 4 Conclusion

This study developed and demonstrated a VMM‐based formulation for LCD 3D printing, achieving key advancements in PDCs. These include:

i) Enhanced Mechanical Performance: The VMM‐based ceramics achieved compressive strengths ranging from 1 to 17 MPa, ceramic densities between 1.8 and 1.9 g cm^−^^3^, and lattice densities between 0.72 and 0.76 g cm^−^^3^. Positioned competitively on the Ashby chart with a strength‐to‐weight ratio between the 10^3^ and 10⁴ J g^−1^ lines, these ceramics are suitable for high‐load structural applications.
ii) Scalable and Intricate Geometries: The low‐viscosity formulation (25.5 ± 0.5 cP) enabled the fabrication of intricate and scalable geometries, including complex structures such as gyroids, stochastic lattices, and graded architectures with tailored feature thicknesses.
iii) Porosity‐Free Ceramics with High‐Temperature Survivability: VMM‐based ceramics retained structural integrity after 2 h of exposure to 1500 °C, outperforming SPR‐based counterparts. This exceptional survivability makes them ideal for extreme environments, such as heat shields, thermal barriers, and microelectronics.

The elemental composition analysis further underscores the advantages of VMM‐010 formulations, which exhibited higher Si content and lower free carbon compared to SPR‐684, resulting in enhanced ceramic densification and interatomic bonding during pyrolysis. These attributes directly contribute to the superior mechanical and thermal properties of VMM‐based ceramics.

CT scans of graded gyroid structures emphasize the quality and help explain the robustness of the VMM‐based lattices. Unlike SPR‐based ceramics, which developed significant voids at feature thicknesses exceeding 1.5‐2 mm, VMM‐based ceramics remained essentially void‐free at the resolution of micro‐CT across all thicknesses. This reliability in producing dense, ceramic features makes the VMM formulation particularly suitable for applications demanding intricate geometries, high structural fidelity, and enhanced energy absorption, such as filters, lightweight structural components, and crash mitigation structures.

Beyond these achievements, this study highlights broader opportunities for VMM‐based formulations:

i) Compatibility with Low‐cost 3D Printers: A notable aspect of this work is demonstrating the compatibility of VMM‐010‐based formulations with low‐cost LCD 3D printers, which operate at lower light intensities. This reduces the entry cost for PDC fabrication and broadens access to advanced ceramic manufacturing technologies.
ii) Sustainable Manufacturing: The integration of recyclable materials and by‐products into ceramic formulations presents a promising avenue for sustainable production. Advances in recyclable slurry systems and organic‐inorganic resins align with global sustainability goals. Combining these strategies with the scalability of LCD 3D printing could reduce the environmental impact while maintaining high performance.
iii) Adaptability for Advanced AM: While techniques like TPP‐DLW excel in micro‐ and nanoscale precision, our work addresses scalability and higher feature thicknesses. The demonstrated ability to produce dense ceramics at these scales expands the utility of PDCs in load‐bearing and high‐temperature applications across aerospace and automotive industries. Additionally, the adaptability of VMM formulations for laser‐based micro‐/nano‐structuring offers exciting opportunities for fabricating high‐resolution ceramic nanostructures for optical, biomedical, and photonic applications.

Optimizing hierarchical or graded lattice designs, and improvements in print quality, offer the potential to further improve stiffness, strength, and energy absorption. Investigating the long‐term durability and failure mechanisms of these ceramics under cyclic loading or harsh environmental conditions would also provide valuable insights for real‐world applications.

The favorable combination of strength, scalability, and versatility offered by VMM‐based formulations and LCD printing offers a promising approach for high‐performance engineering. By addressing challenges in mechanical performance, scalability, and sustainability, this study lays the groundwork for further exploration of material, design, and processing innovations, expanding the potential of PDCs across advanced industrial sectors.

## Conflict of Interest

The authors declare no conflict of interest.

## Author contributions

H.Y.S. contributed to conceptualization; data curation; formal analysis; investigation; methodology; supervision; visualization; roles/writing – original draft; writing – review & editing. V.K. was involved data curation; investigation; writing – review & editing. A.K. contributed to conceptualization; data curation; investigation; roles/writing – original draft; writing – review & editing. A.S.K.: data curation; investigation; writing – review & editing. T.L. participated in conceptualization; data curation; investigation; roles/writing – original draft; writing – review & editing. M.B.J. was responsible for conceptualization; funding acquisition; investigation; methodology; resources; writing – review & editing. B.A. contributed to conceptualization; funding acquisition; resources; writing – review & editing.

## Supporting information



Supporting Information

## Data Availability

The data that support the findings of this study are available from the corresponding author upon reasonable request.
