# Supplementary material for: High‐Performance Polymer‐derived Ceramics in LCD 3D Printing
Source: Adv Sci (Weinh). 2025 Mar 17;12(18):2416176. doi: 10.1002/advs.202416176 (PMC12079501; doi:10.1002/advs.202416176)
Supplement: Supplementary file 1 — Supporting Information [file ADVS-12-2416176-s001.docx]

Supporting Information

**High-Performance Polymer-derived Ceramics in LCD 3D Printing**

H. Yazdani Sarvestani*, V. Karamzadeh, A. Kulkarni, A. Sohrabi-Kashani, T. Lacelle, M.B. Jakubinek, B. Ashrafi

**Table S1.** VVM formulation used in the present work.

| **Component** | **Chemical Structure** | **Role** | **Compositions** | |
| --- | --- | --- | --- | --- |
|  |  |  | **mass (g)** | **%** |
| VMM-010  (Gelest Inc., USA) | 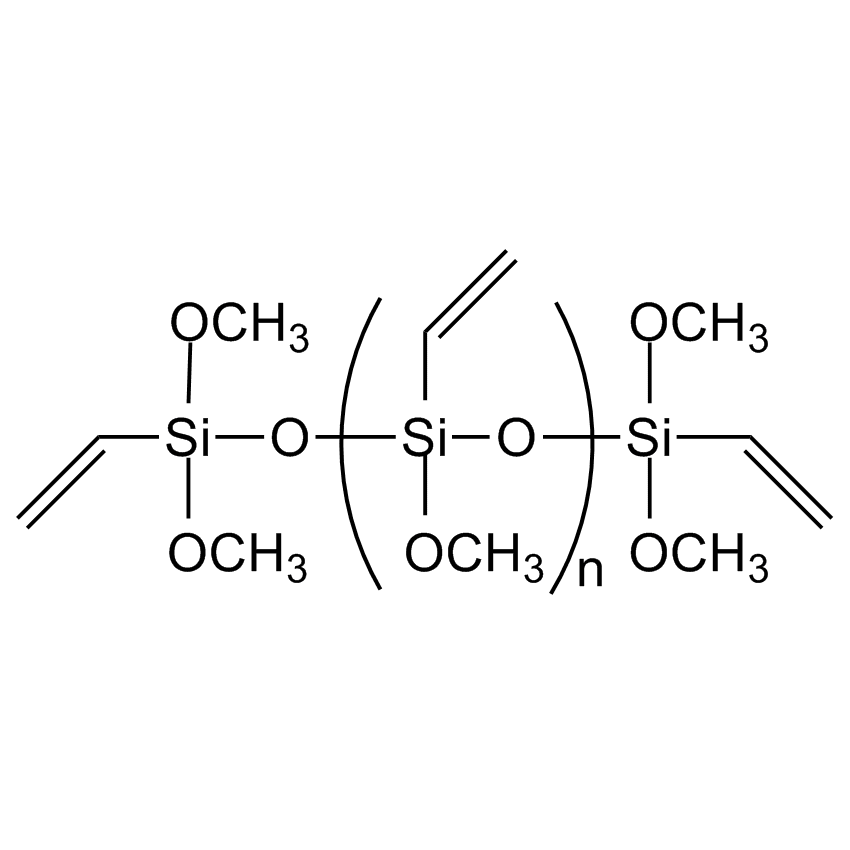  Poly(siloxane) copolymer containing hydride, methyl, vinyl and phenyl groups | Preceramic polymer/Crosslinker | 129.16 | 49.58 |
| SMS-992  (Gelest Inc., USA) | Poly(mercaptopropylmethylsiloxane) | Preceramic polymer/Crosslinker | 129.16 | 49.58 |
| TPO  (Sigma-Aldrich, Inc., Germany) | 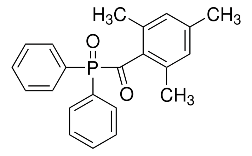  Ethyl (2,4,6-trimethylbenzoyl) phenylphosphinate | Photoinitiator | 1.291 | 0.50 |
| NPS  (Tokyo Chemical Industry Co., Japan) |   2-Nitrophenyl phenyl sulfide | Photoabsorber | 0.5 | 0.20 |
| Pyrogallol  (Molekula Americas LLC, USA) |   1,2,3-trihydroxybenzene | Inhibitor | 0.3876 | 0.14 |
| Filler | - | Filler | 0 | 0.0 |

**Table S2.** SPR formulation used in the present work.

| **Component** | **Chemical Structure** | **Role** | **Compositions** | |
| --- | --- | --- | --- | --- |
|  |  |  | **mass (g)** | **%** |
| SPR-684  (Starfire Systems, USA) |   Poly(siloxane) copolymer containing hydride, methyl, vinyl and phenyl groups | Preceramic polymer | 173.25 | 69.3 |
| SMS-992  (Gelest Inc., USA) | Poly(mercaptopropylmethylsiloxane) | Crosslinker | 75 | 28.0 |
| TPO-L  (Oakwood Products, Inc., USA) |   Ethyl (2,4,6-trimethylbenzoyl) phenylphosphinate | Photoinitiator | 1.25 | 0.5 |
| NPS  (Tokyo Chemical Industry Co., Japan) |   2-Nitrophenyl phenyl sulfide | Photoabsorber | 0.25 | 0.1 |
| Pyrogallol  (Molekula Americas LLC, USA) |   1,2,3-trihydroxybenzene | Inhibitor | 0.4 | 0.1 |
| Filler | - | Filler | 0 | 0.0 |
